# Opportunities and challenges for introduction of balanced energy protein supplementation through routine antenatal care

**DOI:** 10.1371/journal.pone.0361896

**Published:** 2026-11-04

**Authors:** Ruth Waithira, Habtamu Fekadu, Joseph Gaithuma, Justine A. Kavle

**Affiliations:** 1 Kavle Consulting, Gigiri, Nairobi, Kenya; 2 Save the Children US, Washington, D.C., United States of America; 3 Kavle Consulting, Washington, D.C., United States of America; University for Development Studies, GHANA

## Abstract

**Background:**

The World Health Organization (WHO) recommends balanced energy and protein (BEP) supplementation during pregnancy in contexts where prevalence of maternal underweight, defined as body mass index (BMI) < 18.5 kg/m2, is ≥ 20%. Evidence shows that BEP supplementation in underweight pregnant women reduces the risk of low birthweight, small for gestational age, and preterm birth. Yet little is known about how to introduce BEP through routine antenatal care (ANC). This paper explores challenges, opportunities, and recommendations for successful introduction of BEP supplementation through ANC service delivery.

**Methods:**

From August – December 2023, a mixed-methods landscape analysis was carried out, which comprised of a review of country antenatal care guidelines, as well as key informant interviews (KIIs) with thirteen global and country stakeholders. KIIs were recorded, transcribed, and analyzed by themes and subthemes using Dedoose qualitative software 10.0.

**Results:**

Findings revealed confusion around the definition of BEP, coupled with low awareness of the WHO ANC guideline on BEP supplementation among country stakeholders. Provider capacity for BEP provision is weak. Sharing, selling, and stockouts of BEP supplies are common. Data on the feasibility and cost-effectiveness of the introduction of BEP at country level is critical to inform implementation.

**Conclusion:**

Successful introduction of BEP as part of ANC service delivery requires country program implementers to consider government commitments, health provider training, demand generation, compliance, supplies and logistics, local production, and routine monitoring of BEP indicators. Globally, standardized BEP guidance, indicators, and definitions are critical to support quality country implementation. For sustainability, emerging concerns around reductions in global health funding should be addressed in future program design and implementation.

## Introduction

During pregnancy, there is an increased demand for energy, essential amino acids (i.e., protein), and micronutrients for optimal fetal growth and development, and to reduce the risk of poor birth outcomes [1–3]. In nutritionally vulnerable populations, particularly contexts that face widespread food insecurity, underweight pregnant women, defined as body mass index (BMI) < 18.5 kg/m2, are at higher risk of delivering preterm, low birth weight (LBW), and small-for-gestational age (SGA) infants [1,4–6]. Globally, countries severely impacted by the ongoing food and nutrition crises experienced a 25% increase in acute malnutrition among pregnant and breastfeeding women—which rose from 5.5 to 6.9 million [7].

The World Health Organization (WHO) recommends balanced energy and protein (BEP) supplementation for pregnant women in undernourished populations (i.e., settings where ≥ 20% of women are underweight) to reduce the risk of stillbirths and SGA, as part of antenatal care (ANC) services [3]. BEP is defined as a food or supplement product designed to meet a minimum nutrition profile in which protein makes up less than 25% of the total energy content [8]. As an outcome of a BEP Expert Consultation, the recommended composition of BEP was put forth as 250–500 kilocalories of energy, 14–18 grams of protein, and 3–33 grams of fat [9] [the % of fat converted to grams [10].

Early systematic reviews, which formed the basis of the WHO ANC 2016 guidelines, revealed that antenatal BEP supplementation reduces the risk of SGA, stillbirth and LBW [11–13]. Similarly, a 2021 systematic review which compiled data from 15 randomized controlled and quasi-experimental trials showed that antenatal BEP supplementation reduced SGA by 29%, LBW by 40%, and stillbirth by 61% in 9 low- and middle-income countries (LMICs) [14]. Importantly, the impact of antenatal BEP supplementation is greater among malnourished women compared to adequately nourished women across several research studies [11].

Despite the positive effect of BEP supplementation on maternal and infant outcomes, key gaps persist around the optimal dosage and duration of BEP supplementation, targeting strategies and delivery platforms, as well as feasibility, acceptability, and cost-effectiveness of delivery of BEP at scale [15–17]. To date, BEP supplementation has often been limited to humanitarian settings where most countries, especially in sub-Saharan Africa, deliver BEP through existing community management of acute malnutrition (CMAM) programs [15].

Globally, 64% of pregnant women attend at least four ANC visits, which points to ANC as a viable platform for BEP supplementation [3]. Provision of BEP delivered through routine ANC is perceived to increase service uptake [15], yet experience with delivering BEP through facility- and community-based ANC platforms is limited. Few countries have implemented BEP through ANC in development contexts. To gain a better understanding of delivery of BEP supplementation through ANC, we conducted a mixed-methods landscape analysis on challenges and opportunities for BEP introduction across country enabling environments, demand generation, supplies and logistics, service readiness, and monitoring within selected United States Agency for International Development (USAID) country programs.

### Key messages

1. ANC is a viable platform for delivering BEP. Yet, implementation is limited by lack of a standardized definition of BEP, low government prioritization, gaps in provider capacity, and supply shortages.
2. Emerging issues around demand generation and compliance (i.e., sharing and selling of BEP) require targeted social behavior change (SBC) strategies such as provider counselling, leveraging existing platforms, and addressing social norms.
3. Data on the feasibility of BEP introduction through ANC, burden of underweight during pregnancy, and cost-effectiveness of locally produced BEP are needed to guide country action.

### Objectives

1. To ascertain the potential of ANC as a platform for BEP supplementation
2. To identify key challenges and opportunities for the introduction of BEP supplementation through ANC platforms
3. To discuss recommendations to introduce BEP supplementation within ANC service delivery

## Methods

### Study design

From August to December 2023, a mixed-method landscape analysis was carried out by Kavle Consulting in partnership with the United States Agency for International Development (USAID) MOMENTUM Country and Global Leadership (MCGL) project and Save the Children US. Thirteen semi-structured key informant interviews (KIIs) were conducted with global and country stakeholders working in maternal health and nutrition programs. In addition, a review of national ANC guidelines from six countries (i.e., Colombia, Ethiopia, Ghana, Malawi, Mozambique, and Nepal) was carried out to complement data from the KIIs. *Ethics statement:* This mixed-methods landscape analysis was commissioned and approved by USAID MCGL to inform the implementation of United States government-funded programs that provide BEP via maternal health programs*.* Ethics committee approval was not sought, given that the USAID-funded project – MCGL falls under Exemption Category 5 of the U.S. Federal Policy for the Protection of Human Subjects (Common Rule). This exemption from standard institutional review applies as the MCGL project was funded and supported by a United States federal agency (i.e., USAID) to examine a public benefit program. While formal institutional review board (IRB) approval was not required, ethical guidelines and principles, inclusive of verbal informed consent, and data anonymization, were strictly followed. KIIs posed minimal risk – as no medical or sensitive personal information for individual participants was collected. All information was stored securely on an encrypted, password-protected file. All participants’ identifiers were removed prior to data entry and analysis.

### Participant sampling and recruitment

Country selection was based on USAID-funded programming that had maternal health program components and/or inclusion of aspects of BEP implementation within development and/or emergency contexts in Colombia, Ethiopia, Ghana, Malawi, Mozambique, and Nepal. Stakeholders were identified and selected via purposive sampling from USAID MCGL and Save the Children country programs. Stakeholders who worked in maternal health programs and/or who had knowledge of BEP programming or research were selected from governments, international non-governmental organizations (INGOs), multilateral organizations and donors. Balanced representation across these organizations was ensured. Data saturation was reached for this landscape analysis.

### Data collection

A semi-structured interview guide was developed to obtain stakeholder perspectives on the introduction of BEP supplementation via facility- and community-based ANC services. Key themes explored were enabling environment, service readiness, BEP product supplies and logistics, and monitoring (Fig 1). Each KII was 1.5 to 2 hours in duration. All KIIs were conducted online via Microsoft Teams, with at least 2 members of the research team in English and Portuguese. KIIs were recorded and transcribed verbatim in English or from local languages (i.e., Portuguese) to English and subsequently back translated for quality assurance. Transcripts incorporated detailed notes taken during interviews and were reviewed against original audio recordings by the research team to confirm accuracy before analysis.

**Fig 1:**
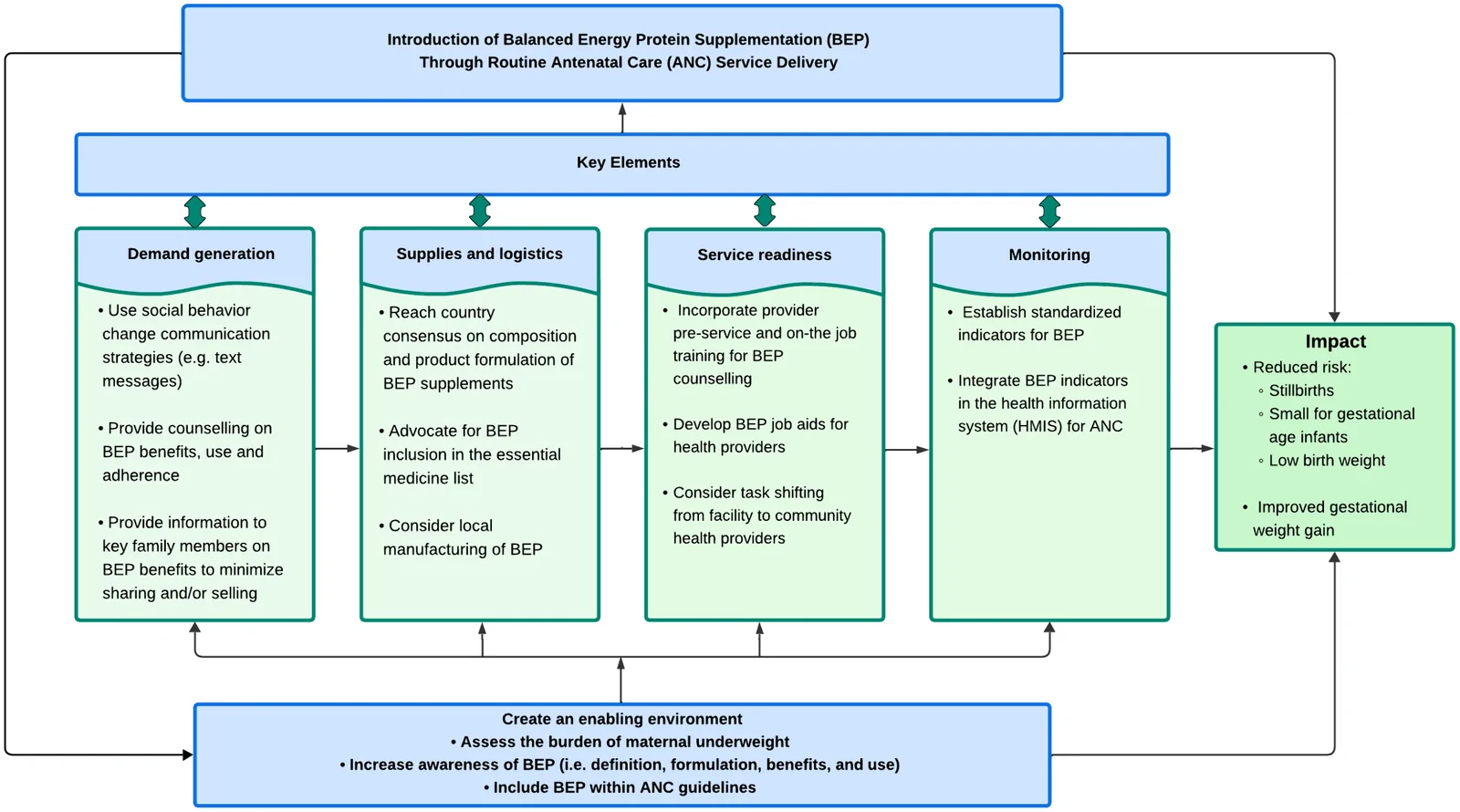
Conceptual framework for country introduction of BEP supplementation via ANC.

### Data analysis

Three research team members collaboratively developed a codebook according to key themes delineated in the conceptual framework (Fig 1). Coding was deductive, based on the predetermined codes. Then, the codebook was refined iteratively to include any emergent themes and subthemes emanating from the data. Themes and sub-themes were analyzed using Dedoose qualitative software version 10.0. Team members independently analyzed the transcripts. Any initial code discrepancies were resolved early in the data analysis process via consensus, and revised codes were applied to the dataset

## Results

### Countries’ readiness to deliver BEP supplementation via the ANC platform

Our findings revealed that three countries, namely Ethiopia, Malawi, and Mozambique, have created an enabling environment (i.e., revised national ANC guidelines) to support the introduction of BEP supplementation. In 2022, Ethiopia revised the national ANC guidelines to align with WHO ANC recommendations – specifically on the identification of pregnant women with protein-energy malnutrition at every ANC contact based on a mid-upper arm circumference (MUAC) cutoff of < 23 cm, and supplementation with corn-soy blend (CSB) – a common BEP product in-country. Similarly, in Malawi, the government revised the national ANC guidelines, ensuring BEP was reflected as a key nutrition intervention delivered during pregnancy, alongside targeted nutrition education, and counselling, as shown in the quote below.

*“The government revised the ANC guidelines to align with the WHO guidance and included BEP as part of the key nutrition interventions for pregnant women* – *alongside nutrition education and counselling on increased energy and protein intake, using culturally acceptable and locally available foods to reduce the risk of stillbirths and small-for-gestational-age neonates.”*

- NGO stakeholder, Malawi

Further, Mozambique has revised the national ANC guidelines to include nutritional assessment and management for underweight pregnant women, which in part aligns with WHO ANC recommendations. While pregnant women are routinely screened during facility ANC consultations, treatment for acute malnutrition using CSB is carried out separately through the Government of Mozambique’s Program in Nutrition Rehabilitation (PRN), which has raised concerns on “how” to integrate BEP, as illustrated below.

*“We have one book for registering women for ANC. Pregnant women are screened during ANC [using MUAC] and then treated for acute malnutrition using CSB via the PRN program, registered in the PRN book and tracked during their postnatal visits. ANC and PRN are considered separate, parallel programs, and the indicators for BEP are not in the ANC book or the ANC guidelines. The Ministry of Health has revised ANC guidelines, but I don’t know if the nutrition program can integrate [BEP]. We do not have nutritionists in health clinics, but we have nurses* – *so it is an opportunity to include BEP in ANC and build their capac*ity.”

– NGO stakeholder, Mozambique

### Key challenges and opportunities for the introduction of BEP supplementation through ANC

#### Lack of standardized BEP definition and low awareness of recommendations on BEP.

Country and global stakeholders held conflicting understandings of the definition of BEP in terms of use and composition. Some country stakeholders confused BEP with ready-to-use therapeutic foods (RUTF) and small-quantity lipid-based nutrition supplements (SQ-LNS) for treatment of child wasting. A global stakeholder felt that this uncertainty surrounding BEP stemmed from the lack of a standardized definition of BEP in the WHO 2016 ANC guidelines, as expressed in this quote.

*“If you look at the [WHO] guideline, it’s not clearly defined in terms of what the exact composition of BEP is….. so, we refer to the current literature, which sets protein at less than 30% of total energy intake. That’s the definition we use, and other organizations are probably using the same definition, but it is not from WHO.”*

*-* Multilateral organization stakeholder

Moreover, a few country stakeholders conveyed limited awareness of BEP as part of the WHO ANC guideline and low country prioritization of BEP in national policies and guidelines on maternal health, despite BEP being implemented as part of emergency programming in some contexts, such as Colombia, described below.

*“No, I’m not sure I have read it [BEP recommendations in the WHO 2016 guidelines]. Health providers are also not aware of these [WHO] recommendations, because it’s not common to use BEP within the health system. The government [does not] have policies or guidelines to facilitate the introduction of BEP supplements via ANC platforms.”*

*—* NGO stakeholder, Colombia

### Gaps in provider capacity limit provision of BEP

Provision of BEP as a component of ANC, is largely absent in developmental contexts – even in settings where maternal underweight is a country priority, as described below.

*“We have not as a country operationalized BEP supplementation, but it is part of the guidelines for ANC. There are opportunities in our guidelines for the community-based management of acute malnutrition through which the government conducted community nutrition screenings and provided CSB mixed with vegetable oil to any pregnant or lactating woman with a MUAC of less than 22 centimeters. We have not identified CSB as a BEP product, but the opportunity is there.”*

*-* NGO stakeholder, Malawi

To introduce BEP through ANC, stakeholders unanimously emphasized the importance of training and equipping facility and community providers with job aids. Some global stakeholders noted that maternal nutrition training within ANC service delivery has been neglected, emphasizing the need for pre-service and on-the-job training to support quality counselling during pregnancy, as expressed below.

*“It is important to ensure that there are capacity building activities as well as proper training to provide or support quality counseling [for BEP].”*

*-* Global donor organization stakeholder

Across all six countries, stakeholders recognized task shifting from facility health providers to community health providers as a crucial need to “*increase accessibility to various ANC services such as weighing pregnant mothers, checking height and MUAC measurements, and distribution of food supplements*,” while balancing job responsibilities and provider workload.

### Challenges with BEP supplies: Formulation, distribution, storage, and cost

In Colombia, Ethiopia, and Mozambique, stakeholders reported that BEP is primarily supplied through parallel systems, via CMAM and integrated management of acute malnutrition (IMAM) emergency programming. In Ethiopia and Mozambique, stakeholders noted that stockouts of maternal nutrition commodities such as BEP were a common issue with parallel supply systems, as relayed in this quote.

*“We may have to create a parallel system during the testing process [for the introduction of BEP]. Currently, we have parallel systems in the supply chain for some nutrition commodities provided through the government – but local stockouts are common.”*

*—* NGO stakeholder, Ethiopia

To address this, country stakeholders emphasized the need to include BEP supplements in the essential medicine list for prioritization within country governments’ supply chains. Some country and global stakeholders also expressed uncertainty about whether BEP supplements should be considered as food or medicine. This classification was noted to vary by countries depending on the type of BEP formulation (i.e., powdered supplements, fortified biscuits, fortified spread, corn-soy blend supplement, or supplemental beverages). BEP is also considered “*bulky*, *which can result in difficulties for its inclusion in the government supply chains and efficient distribution and delivery at the country level,”* according to a global stakeholder.

In Malawi, a country stakeholder reflected on how withdrawal of donor support discontinued previous supplementary feeding programs for underweight pregnant and lactating women, which raised concerns about the long-term sustainability of BEP programming without dedicated financing mechanisms. The majority of stakeholders emphasized that future BEP initiatives require early transition and sustainability planning to mitigate disruptions in the event of withdrawal of external support.

Further, the potential high cost of importing BEP supplements was relayed by nearly all country stakeholders, who noted that local BEP production could ease storage and transportation constraints, as well as reduce costs. In Malawi and Mozambique, stakeholders described potential low government acceptance of BEP supplementation by the Ministries of Health and emphasized the need for on-the-ground assessments to better inform whether local production versus global procurement of BEP is feasible and appropriate, as explained below.

*“There needs to be processes of how we can integrate [BEP] into the existing supply chain and management systems within the Ministry of Health. [Depending on the formula], it is difficult to integrate bulky nutrition commodities* – *corn-soy blend and vegetable oil are both bulky. Because BEP will be recognized as food, there has to be a lot of engagement and consultations on how [BEP] can get on the essential supplies list for the Ministry of Health.”*

*–* NGO stakeholder, Malawi

Some country stakeholders expressed concerns that local production may involve additional costs due to importation of key ingredients, which may not “*necessarily lead to greater affordability and availability of locally manufactured BEP*.” A global stakeholder emphasized the need for data on the cost-effectiveness of integrating BEP into the package of maternal health interventions delivered during pregnancy, as relayed in this quote.

*“This is an opportunity to work with donors. We expect some cost-effectiveness work or operational research to accompany BEP programming to understand the costs involved, and to set the stage for future discussions with governments on scaling the program.”*

— Global donor organization stakeholder

### Demand for BEP: acceptability, compliance, sharing and selling

Most global and country stakeholders highlighted the importance of generating demand for BEP to support compliance. Stakeholders from Malawi and Ethiopia raised concerns about the affordability, accessibility, quality, and cultural relevance of BEP supplements selected for country-level implementation. All country stakeholders also relayed that widespread cultural food practices, including strong social norms around eating together as a family, may affect individual consumption of BEP supplements. In addition, the majority of global and country stakeholders emphasized the importance of sensory characteristics such as taste, texture, and smell, as well as the use of familiar, culturally acceptable, and locally available food to promote BEP acceptability and compliance, as explained in the quote below.

*“Successful integration of BEP into ANC services requires a deep understanding of the cultural, nutrition, and logistical landscape. Before introducing a new supplement, it is essential to assess women’s attitudes and knowledge about supplements* – *factors like accessibility, affordability, and potential allergens should be examined, especially in relation to the population’s needs, to guide decision-making. It is not enough to focus solely on protein quantity and nutritional quality; bioavailability of the supplement matters significantly. We should explore locally available and culturally accepted protein and energy sources and consider alternatives that align with available resources.”*

*—* Global donor organization stakeholder

Country and global stakeholders widely identified inadequate ANC counselling as a key challenge to women’s understanding of the importance of BEP, appropriate use, and adherence*.* Most country and global stakeholders also described household sharing of BEP supplements as a hindrance to compliance. These stakeholders explained that pregnant women are often expected to share supplements with other family members due to household food insecurity, competition for food, and the perception that limited food resources should be prioritized for children, as described by a global donor stakeholder.

*“What is difficult is that when you give [supplements] to pregnant women, the main issue is not that they don’t want to eat it* – *there is too much competition within the household, and the feeling that [the supplement] needs to be given to the children. If it’s a good product and people see it works, then competition increases in the household because there’s not much [to eat], and there is limited understanding that pregnant women need to be supported and nourished.”*

*—* Global donor organization stakeholder

Some stakeholders recommended to “*label BEP as medicine,”* and clarify that BEP is “*only for pregnant women”* during ANC counselling to minimize the risk of household sharing with family members. Further, in Ethiopia, one stakeholder noted the widespread selling of supplementary foods designated for pregnant women in the management of acute malnutrition within HIV-nutrition-integrated programs, which was identified as a potential issue for BEP supplementation within ANC services. The women often sold the BEP supplements provided to them because they did not like the taste or eating the same supplement daily. Mothers also complained that the supplements were “*too heavy”* to transport in large quantities, as detailed below.

“*When providing BEP supplements, it is a challenge, as [women] may not like the taste and they get bored with eating the same supplement. These commodities are also very heavy, and when provided for 2 months, it’s a lot to carry home, so they sell it.”*

— NGO stakeholder, Ethiopia

### Weak monitoring of BEP supplementation via routine health systems

In countries with limited data on the prevalence of underweight pregnant and lactating women, the need to strengthen data management and monitoring systems was echoed by all stakeholders. A majority of country stakeholders proposed utilizing facility registers at service delivery points with a clear reporting flow from sub-national to national levels to improve data management and monitoring systems for BEP. Yet, no country examined in this landscape analysis routinely collects data on BEP via health systems. The need to include BEP indicators as part of routine health information systems to monitor product distribution and program impact was described by most stakeholders, as relayed in the quote below.

“*Currently, we capture antenatal care data in our health information system, including registers for different components of antenatal care such as nutrition counseling and iron and folic acid supplementation. If we were to introduce BEP supplementation via ANC, we would need to integrate BEP indicators into our existing registers and reporting system.”*

— Government stakeholder, Ghana

## Discussion

This landscape analysis reveals ANC is a viable platform for delivery of BEP in development contexts. Our findings showed that Ethiopia, Malawi, and Mozambique have revised national ANC guidelines based on the 2016 WHO ANC guideline recommendations. These updates create an enabling environment for the introduction of BEP supplementation through the ANC platform. Yet a conflicting understanding of the composition and use of BEP, lack of a standardized definition of BEP, and low awareness of BEP among most country stakeholders may impede the introduction of BEP supplementation through ANC. Limited health provider capacity and training, sharing, and selling of BEP supplements, as well as stockouts and the potential high cost of importing BEP supplies were primary concerns across countries included in this analysis. Insights from stakeholders indicated that monitoring of BEP supplementation via routine health systems is weak, which is further exacerbated by limited data on underweight pregnant women at the country level.

### Government commitment: Prioritization of maternal underweight

While maternal undernutrition is increasingly recognized as an important issue for countries in this landscape analysis, screening and treatment of moderate and severe child wasting has been primarily prioritized [18,19]. Moreover, lack of country data on the prevalence of underweight pregnant women has hindered the prioritization and targeting of undernourished women during pregnancy with BEP. Our findings are consistent with other evidence, which reports significant data gaps for maternal nutrition – given routine tracking systems do not adequately monitor either healthy or insufficient weight gain during pregnancy [6,20–22]. Another critical issue is that national household surveys, such as Demographic and Health Surveys- a key source of data on maternal health and nutrition – have historically only collected data on underweight for women of reproductive age and not pregnant women.

### Demand creation through targeted SBC tools

This landscape analysis revealed limited understanding among health providers, community members, and pregnant women themselves about the composition of BEP, “*wha*t” BEP is used for and “*how*” BEP differs from treatments for child wasting, which can negatively affect community acceptability of BEP. Similarly, recent evidence from rural Bangladesh shows that health providers, pregnant women, their husbands, and mothers-in-law were unfamiliar with BEP as a new product and were hesitant to use BEP. In Bangladesh, acceptability was further hindered by uncertainty about the effects of BEP (i.e., what will happen to women if they take it), and the added value of BEP- especially when compared with a locally available high-protein drink (i.e., Mother Horlicks – Unilever) – marketed to improve nutrition during pregnancy [16]. Together, these findings highlight the need for demand generation for BEP through provider counselling to improve understanding and address misperceptions (i.e., BEP use leads to childbirth complications) [16]. Leveraging existing platforms such as group ANC and mother-to-mother support groups can improve understanding and acceptability of BEP, as well as dietary intake during pregnancy [23,24]. In addition, mobile text messaging can also drive demand through daily reminders to consume BEP alongside nutrition information on the benefits and use of BEP, and as a means to address misinformation [15,25,26].

### BEP supplies, logistics and local production

BEP supplementation has been primarily delivered via vertical humanitarian programming, and not through development contexts. Lessons from emergency programming show that disruptions of supplies can undermine trust in nutrition-health programming and weaken adherence when BEP supplements are not reliably available [15]. Our findings revealed that local BEP production was perceived as a viable strategy to not only increase in-country BEP availability but also improve sustainability – in terms of community acceptance. These findings were echoed by data from Ethiopia, which reported greater acceptability and use of locally produced BEP made of corn and soybeans blended flour in comparison to imported BEP [27]. Similarly, in Bangladesh, two research studies found high acceptability of domestically manufactured BEP made from locally available, affordable, and culturally acceptable foods (i.e., pigeon peas, bananas, peanuts, sesame seeds, whole milk powder; puffed rice, lentil powder) [28,29]. For local manufacturing of BEP to be viable, quality-assurance mechanisms should be established to ensure that supplements are manufactured, packaged, and stored in controlled and uncontaminated environments [3]. There is also a need for country-specific assessments to determine the feasibility of local BEP production.

Consistent with stakeholders’ emphasis on culturally acceptable BEP formulations, the use of locally available foods and recipes as BEP formulations that meet nutrition recommendations is another potential approach to ensure country level sustainability [29]. In Bangladesh, a village-prepared cereal-pulse mixture with oil distributed via community nutrition centers provided pregnant women with 600 kcal/day to increase weight gain during pregnancy and reduce low birth weight [30]. Further work is needed on cost-effectiveness and quality assurance mechanisms to ensure that locally developed formulations meet recommended BEP composition standards [9].

### Improving BEP coverage: Targeting vs blanket approach

Concerns regarding the financial resource implications of the introduction of BEP into ANC emphasized the need to examine targeting vs blanket approaches in terms of cost effectiveness, training needs, and delivery efficiency. Existing evidence shows that targeting criteria applied at a household level have prioritized food insecure populations, internally displaced persons, marginalized clans, or at an individual level based on nutritional status [15,17]. Targeting BEP based on low maternal BMI or MUAC is considered safe, affordable, and impactful [31,32], yet does not account for inadequate gestational weight gain —a key criterion that may identify a greater number of at-risk pregnant women [17]. Consideration of how communities perceive targeting criteria is critical, as negative perceptions may impact understanding and acceptability of the targeting approach. In northern Bangladesh, pregnant women did not accept BEP targeting criteria based on body weight because some felt excluded based on a determinant that was out of women’s control. Instead, pregnant women and other community members preferred other criteria, such as household poverty and diet quality (i.e., variety, balance and adequacy) [32]. Targeting approaches should not hold the assumption that low household food availability is the primary cause for low dietary intake among pregnant women as other factors, such as poor appetite, nausea, infection, and deliberately reducing meal intakes to avoid delivery complications, also play a role [33,34]. On the other hand, this landscape analysis underscored that blanket supplementation in development contexts may increase risk of overweight and obesity in non-vulnerable populations.

### The need to address compliance: Sharing and selling of BEP

Emerging concerns around BEP adherence were attributed to household sharing of BEP supplements, due to competition for food, and prevailing social norms that prioritize young children when food is scarce. Similarly, recent data from Ethiopia revealed that 57% of pregnant women shared BEP with children and husbands, as eating alone was a “*taboo*”, and mothers did not want their children to become malnourished [27]. A recent global review also echoed that cultural norms and food insecurity dictated household sharing of meals, which can exacerbate BEP sharing and undermine compliance in Afghanistan, Bangladesh, Chad, Ethiopia, Kenya, Madagascar, South Sudan, and Syria [15]. In Bangladesh and Nepal, studies tracking food supplement consumption by family members revealed that sharing occurred only in the first week of a two-week trial period for daily BEP supplementation [29,35]. In the Bangladesh study, additional packets were provided at an initial visit to accommodate household family members’ ability to taste and approve the supplement and ensure that women have enough BEP product for daily use [29]. However, in programmatic settings, procurement, and distribution of additional supplements for family members would likely not be feasible nor cost effective. These findings reinforce our landscape analysis recommendations that stronger ANC counselling and clear messaging that BEP is intended only for pregnant women can reduce household sharing. Targeted counselling, and proper labelling and prescription of BEP as “*medicine*,” as voiced by stakeholders, is critical for correct BEP use and adherence throughout pregnancy [27]. There is a need to further examine how sharing should be addressed and minimized within the context of cultural and social norms.

Selling BEP was a significant concern in countries where the sale of other therapeutic supplements (i.e., RUTF) was reportedly common- such as in Ethiopia. The drive to sell rather than consume BEP was attributed to the monotony of consuming the same supplement daily, the bulkiness of large quantities of BEP supplements required during pregnancy, and prioritization of families’ financial needs to purchase household food items. In Nigeria and Uganda, mothers similarly sold RUTF intended for their children in local markets and mobile money kiosks due to economic hardship and the need to generate household income [36,37]. In Nigeria, local investigations and civil society groups revealed that mothers were coaching their children to “*act sick*” to access RUTF for mothers to sell in local markets [36]. Monitoring BEP regulations that prohibit resale, and social safety net programs for vulnerable households can discourage inappropriate use of BEP.

Future programs should examine how to reduce the sale of BEP supplements through targeted SBC, inclusive of community sensitization, and counselling on the intended use of BEP taking into account cultural and social norms. While both selling and sharing behaviors are not limited to BEP – similar practices have been observed with therapeutic, supplementary, and fortified blended foods provided to adults and children – which warrant further investigation – given the potential to undermine the effectiveness and impact of BEP programming long term.

### Emerging concerns for sustainable introduction of BEP via the ANC platform

A key component in the sustainability of BEP programming is reduced dependence on volatile donor funding [27]. Yet, concerns regarding the costs of procuring and importing BEP supplements, as well as the sustainability of supply chains without dedicated funding mechanisms, were recurrent themes in this landscape analysis. These findings have become increasingly relevant in light of the 2025 reductions in global health funding of 27 billion dollars by the United States Government, which have disrupted maternal and child health services inclusive of routine ANC [38]. In a fragile aid environment, the question of how to sustainably finance BEP supplementation becomes increasingly urgent – given the previous reliance on external donor funding and competing priorities facing country governments with limited budgets allocated to nutrition and health interventions [39].

Funding concerns from this landscape analysis reflect existing evidence on the impact of reduced global health investments on national health systems required for sustainable introduction of BEP in routine maternal health service delivery. Projections estimate that funding reductions will constrain health service delivery for 16.8 million pregnant women and 11.3 million newborns annually. By 2040, an increase in maternal mortality (+29%), under-five mortality (23%), and stillbirths (13%) may translate into an additional 510,000 maternal deaths, 7.9 million child deaths, and 1.8 million stillbirths [40]. WHO and civil society organizations have documented widespread health facility services suspensions, stockouts of nutrition supplements, and reductions in the community health workforce across LMICs [40,41], due to dramatic decreases in health workforce training [40]. Monitoring and evaluation systems have also been negatively affected through reduced investment in routine data collection, reporting, and health information systems – which may impede efforts to integrate BEP indicators into routine health information systems, reduce available data for decision makers to respond effectively, and further weaken accountability in maternal and child health programs [42,43].

### Strengths and limitations of the landscape analysis

This mixed-methods landscape analysis offered diverse yet valuable insights across six countries in sub-Saharan Africa and South Asia, and a comparative view of the current policy environments, challenges, and opportunities in the introduction of BEP via ANC. By combining key informant interviews with a review of country ANC guidelines, the analysis captured both the current realities described by global and country stakeholders, and the formal policy landscape. Findings of this landscape analysis can also provide key insights into how countries can address the introduction of BEP supplementation through ANC platforms.

This mixed-method landscape analysis had several limitations. This analysis was not intended to be exhaustive given the short timeframe for implementation and donor mandate- which limited the analysis to USAID country programs with maternal health and/or nutrition components. The analysis incorporated a variety of stakeholders inclusive of multilateral organizations, implementing partners, and governments. Stakeholders were purposively selected, which may have contributed to selection bias. Yet, this was minimized by inclusion of participants working across different sectors and organizational affiliations. In addition, responses may have been influenced by possible social desirability bias. To mitigate this risk, we used probing techniques to elicit in-depth responses and triangulated stakeholder perspectives with the review of in-country documents, inclusive of maternal health/ ANC guidelines. While country selection was limited to USAID-supported maternal health programs, rich contextual detail on BEP programming and research from various countries, allow for transferability to other contexts. While the analysis did not involve direct interviews with frontline health providers and community health workers, we gleaned perspectives on training, counseling, job aids, and task shifting, which are critical aspects of their role in the introduction of BEP through ANC. Future research should incorporate broader country representation, larger samples, frontline providers, and findings from implementation studies.

### Considerations for introduction of BEP Supplementation through the ANC platform [24]

The necessary actions to introduce BEP supplementation through routine ANC services at the global and country level are outlined below (Table 1). Moving forward, reviewing country-specific data, priorities, policies, and guidelines on underweight among pregnant women is a critical step to ascertain program readiness. To introduce BEP supplementation into routine ANC, country governments and implementation partners should evaluate service readiness, product identification and supply chain, and social behavior change.

**Table 1:** Global and country level considerations for introduction of BEP supplementation through the ANC platform.

Global Level	
**Policy and guidance**	• Standardize global definition of BEP to ensure consistency in product formulation, targeting, and messaging • Create comprehensive global implementation guidance for BEP supplementation, outlining how BEP is provided within the ANC package and is coordinated with other supplementary food interventions [44] • Develop standardized BEP performance indicators for integration within routine health systems to monitor use, distribution and impact, based on existing WHO ANC guidance
**Country Level: Program Readiness and Planning for BEP Introduction**	
**Service readiness**	• Ascertain the national and sub-national burden of underweight pregnant women and adolescents (i.e., ≥ 20%) using existing data or collecting new data in selected areas of the country • Review existing reports and programs to understand how, where, and when to introduce BEP via ANC • Identify implementation platforms, whether as part of ANC services or existing programs, (i.e., supplemental feeding programs like CMAM) • Assess health workforce capacity and delivery approaches for BEP, including task shifting • Ensure provider training includes updated guidance on BEP (i.e., in-service, and pre-service, where possible)
**Product identification and supply chain**	• Gain in-country consensus on the definition and composition of BEP • Determine the most appropriate local products and formulations for underweight pregnant women that meet WHO BEP recommendations • Evaluate BEP product options based on packaging, cost, formulation, storage and transportation, risk of meal replacement or sharing, cultural acceptability, safety, salt and sugar content, nutrient profile, and energy density
**Social behavior change**	• Conduct formative research on community perceptions, beliefs, and practices on BEP supplement use, including how cultural, social, and gender norms may influence consumption of locally produced BEP • Ascertain facility and community providers’ knowledge and attitudes around BEP provision during ANC • Equip health providers with updated counselling skills and materials (i.e., job aids, which can be integrated as part of ANC) • Strengthen demand generation through SBC strategies (i.e., mobile text messaging and targeted counselling) on BEP benefits and use, and to address misinformation
**Future implementation research**	• Review evidence from ongoing clinical trials, when available, on optimal BEP composition, regional fortification, and dietary impact • Examine targeting vs. blanket approaches with regard to training, health service delivery efficiency, cutoffs by MUAC and BMI, and any drawbacks (i.e., increased risk of being overweight/obesity and costs) • Use country program data to document cost-effectiveness, feasibility, and acceptability of delivering BEP through routine ANC, which can support future scale-up • Investigate BEP supplementation during lactation, and its long-term effects on maternal and infant health outcomes in undernourished populations • Explore the linkage between local food production systems and BEP supplementation, including potential to reduce costs and improve in-country availability of BEP products

## Conclusion

Provision of BEP through routine ANC is a promising intervention to improve maternal and neonatal health outcomes. However, successful introduction of BEP into ANC requires country program implementers to understand the opportunities and challenges to BEP service delivery, supply chain, demand generation, SBC, and routine monitoring. Globally, a standardized definition of BEP, standardized operational guidance, and monitoring indicators for routine health information systems are needed to support tailored country implementation of BEP.

In countries that currently provide BEP during routine ANC services (i.e., Philippines), south-to-south learning should be facilitated on inclusion of BEP in country ANC guidelines, demand generation, and local production of BEP. While local production of BEP has been proposed to reduce cost and logistical implications, data on cost-effectiveness and feasibility are sorely needed. Future implementation research and in-country program assessments can provide valuable insights into the implications of targeting vs blanket BEP supplementation approaches in terms of training, cost-effectiveness, and coverage. In sum, by considering the programmatic recommendations outlined in this paper, countries can take steps to enhance the reach, quality, and sustainability of BEP supplementation within ANC platforms, contributing to improved maternal and infant health outcomes.
